# The Know-Do Gap: Understanding and Improving Service Quality Among Pharmacies Providing Injectable Contraceptives Through a Mystery Client Study in Nepal

**DOI:** 10.9745/GHSP-D-21-00657

**Published:** 2022-06-29

**Authors:** Sujan Karki, Margaret Chappell, Ben Johns, Sarah E.K. Bradley

**Affiliations:** a Abt Associates, International Development Division, Kathmandu, Nepal.; b Abt Associates, International Development Division, Rockville, MD, USA.

## Abstract

Nepal is one of the first countries to license pharmacists to administer injectable contraceptives, through Nepal CRS Company's (CRS) Sangini private pharmacy network. There are more than 3,400 Sangini pharmacies in Nepal, including in hard-to-reach mountain areas, where these outlets are a key access point to injectables and other short-acting methods for those who lack access through the public sector or prefer the private sector.

We compared the performance of Sangini pharmacists in (1) CRS-led technical support visits, when providers were aware of being observed, and (2) mystery client visits, when providers were not aware of being observed, to assess any gaps between pharmacists' knowledge and practices. We also assessed how well Sangini providers counseled on injectables and compared counseling on injectables with counseling for oral contraceptives.

We found high levels of adherence to training guidelines on counseling on injectables. However, we identified significant differences between pharmacists' understanding of what they should do and what they actually do in practice, referred to as the know-do gap, in providing privacy, assessing client needs, and determining medical eligibility for hormonal methods. CRS took several steps to narrow the know-do gap through its programming, which may be a useful example for other countries as they expand the role of pharmacies in family planning service provision. Despite highlighting several areas for improvement, the findings show that Sangini providers both know how to and practice appropriate counseling on both injectable and oral contraceptives, suggesting that pharmacists can successfully expand their family planning offerings and equip clients with the information needed to select an appropriate method of their choice.

## Abstract (precis)

Private pharmacists in Nepal CRS Company's Sangini network provided quality counseling on injectable contraceptives to mystery clients, suggesting that pharmacists can successfully expand their family planning offerings and equip clients with the information needed to select an appropriate method of their choice.

## Teaser

**Key Findings:**

- Trained and accredited pharmacists in Nepal are allowed to administer injectable contraceptives. Private pharmacists in Nepal CRS Company's Sangini network provided quality counseling on injectable contraceptives to mystery clients, suggesting that pharmacists can successfully expand their family planning offerings and equip clients with the information needed to select an appropriate method of their choice.
- While Sangini pharmacists met counseling standards, the mystery client study identified know-do gaps in providing privacy, assessing client needs, and determining medical eligibility for hormonal methods.

**Key Implication:**

- Policy makers should consider that private pharmacies such as those in the Sangini network provide a promising opportunity to increase access to injectables and increase women's contraceptive options. Pharmacists are able to adhere to quality standards for counseling on injectables and other contraceptive methods.

## INTRODUCTION

Private providers are an important source of family planning (FP) in low- and middle-income countries; on average across 36 low- and middle-income countries, 1 in 3 modern contraceptive users obtains their method from a private source.^1^ Similarly, in Nepal, the private sector is a key source of modern contraception, and 25% of modern contraceptive users rely on the private sector for their methods. Forty-one percent of oral contraceptive users and 26% of injectable users obtain their method from private sources.^2^

The efficacy of private sector FP services in meeting clients' needs and ultimately improving contraceptive access depends on the quality of these services. Since the foundational Bruce-Jain quality paradigm for international FP was developed in 1990, quality has been a key focus of FP service delivery.^3^^,^^4^ In recent years, the global FP community has transitioned toward a rights-based and client-centered approach to contraceptive service delivery.^5^^,^^6^ These approaches place increased importance on particular quality dimensions, including ensuring that clients have access to a full choice of methods and receive respectful, dignified care. With evidence showing that increased quality of care is associated with both contraceptive uptake and continuation, ensuring and monitoring high-quality FP service delivery is of utmost importance.^7^^–^^10^

Evidence from multiple countries shows that training and supportive supervision can have a positive effect on the quality of FP services. A study in the Philippines found that training FP service providers in information exchange and their supervisors in facilitative supervision significantly improved the quality of care received by clients.^11^ A study in Nigeria found that a training program and supportive supervision activities had a positive effect on the quality of counseling services, especially on the range of contraceptive methods discussed by providers, their interpersonal skills, and overall knowledge.^12^ Few studies have explored this issue in Nepal, but Agha et al. found that clients' perceptions of the quality of services improved after the U.S. Agency for International Development (USAID)-funded Commercial Market Strategies project gave franchisees within a network basic reproductive health training.^13^

Sustaining Health Outcomes through the Private Sector (SHOPS) Plus is USAID's flagship initiative in private sector health. The project seeks to harness the full potential of the private sector and catalyze public-private engagement to improve health outcomes in FP, HIV/AIDS, maternal and child health, and other health areas. SHOPS Plus worked in Nepal from 2016 to 2021 to improve access to and quality of FP service delivery by building the technical capacity and financial sustainability of Nepal CRS Company (CRS), a leading Nepalese social marketing organization and key USAID partner.

Although the modern contraceptive prevalence rate in Nepal is relatively high at 43% among married women, access to contraception is difficult in rural mountain and hill districts with rugged terrain, poorly constructed roads, and very few pharmacies and other health outlets.^14^ To improve contraceptive access in these underserved areas, SHOPS Plus supported CRS to implement its Sangini network, which encompasses nearly 3,400 pharmacies throughout the country. While CRS is broadening access to contraception through its network, Sangini outlets in rural and remote areas are scattered and focused primarily in market centers, meaning that the network is not completely reaching hard-to-reach populations. It is also important to note that although CRS refers to Sangini as a network of pharmacies, generally these pharmacies are not owned or staffed by professional pharmacists; rather, they are run by auxiliary-level health workers consisting primarily of auxiliary health workers and medical assistants, as well as individuals who have received training in drug sales only.^15^ While these outlets are not usually owned or staffed by pharmacists, we refer to them as “pharmacies” in this article because that is the term used by CRS.

Sangini is the first and only private pharmacy network in Nepal licensed to provide overbranded Depo-Provera injectables, the leading short-acting method in the country.^14^ Sangini pharmacies have administered injectables since 1994. The World Health Organization (WHO) has identified task sharing as a promising strategy to address the lack of health care workers trained and licensed to provide FP globally, recommending that pharmacists administer injectable contraceptives.^16^ However, pharmacists in many countries are unable to do so due to regulations that prevent them from providing medical services.^17^ Indeed, few other countries have formally changed their policy to allow pharmacists to provide injectables, although more are taking steps to do so, such as Rwanda in 2020.^18^

Sangini is the first and only private pharmacy network in Nepal licensed to provide Depo-Provera injectables, the leading short-acting method in the country.

As the only private pharmacies licensed to provide injectables in Nepal, Sangini outlets serve as a key access point to injectables for clients who prefer to go to the private sector or who may lack access through the public sector. The outlets also provide oral contraceptives, condoms, and emergency contraception and have referral linkages for providing long-acting reversible and permanent contraceptive methods. CRS provides a substantial share of the country's contraceptives. Indeed, 38% of short-acting method users rely on CRS socially marketed products, including 58% of oral contraceptive users.^1^ Moreover, 11% of injectable users obtain their method from Sangini pharmacies.^14^*

All Sangini network service providers receive a standardized 3-day Sangini training, with an emphasis on injectable administration. After training, for quality assurance and improvement, CRS conducts supportive supervision, or technical support visits (TSV), to service delivery providers in Sangini pharmacies located in 49 of Nepal's 55 rural mountain and hill districts. CRS prioritized these 49 hard-to-reach districts through its USAID-supported Ghar Ghar Maa Swasthya (GGMS) project. The TSV checklist, which was revised by CRS and SHOPS Plus in 2018, measures Sangini provider adherence to several quality protocols, including maintaining their facility (cleanliness, privacy, visibility of information, education, and communication materials), assuring clients of privacy, asking demographic questions to build rapport with the clients (age, parity), asking preference questions (desire for more children, contraceptive preferences), completing medical screening (asking about pregnancy, other medical issues), explaining contraceptive options, and counseling on side effects of the chosen contraceptive and what to do if side effects occur. These protocols were developed in alignment with WHO and Government of Nepal guidance.^19^^,^^20^ CRS holds refresher trainings for providers who are not making sufficient progress toward meeting quality standards, as defined by a poor TSV score.

The effectiveness of these trainings and subsequent TSVs in ensuring the quality of FP service delivery, both in knowledge and practice, has not previously been measured in the Nepal Sangini network. Moreover, we could not find evidence in the existing peer-reviewed literature of any quality assessment of pharmacists trained to provide injectable contraception. To address this gap, improve CRS's training and supervision programs, and provide lessons learned to improve other programs looking to expand the availability of injectable contraception through pharmacies, SHOPS Plus implemented a mystery client survey to address the following research questions:

1. How does the performance of Sangini providers compare when measured during TSVs (when providers are aware that quality assurance officers are observing them) versus mystery client visits (when providers are not aware that they are being observed)?
2. How well do Sangini providers counsel prospective clients on injectables? How does their counseling on injectables, a method that is accessible in Nepalese pharmacies only via the Sangini network, compare to their counseling on oral contraceptives, a method provided by pharmacists around the world?

To improve CRS's training and supervision programs, we implemented a mystery client survey to assess how well pharmacy providers counsel on injectable contraceptives.

We compared the mystery client visit findings with TSV data for the same Sangini facilities, measuring adherence to quality protocols outlined in the TSV checklist. At the end of this article, we describe potential reasons for the findings, comparisons with previous studies, how CRS modified its oversight of the Sangini network in response to our findings, and potential avenues for continued monitoring of the TSVs.

## METHODOLOGY

SHOPS Plus conducted a mystery client (also known as a standardized patient) survey, using trained interviewers posing as pharmacy clients and reporting on their experiences. The target population for the mystery client survey was Sangini network pharmacies with a pharmacist who received at least 1 TSV (described later) from CRS between July 2016 and May 2018. The survey used a stratified multistage sampling approach to select the sample of pharmacies. CRS manages the 49 GGMS districts in 6 supervision areas, each managed by a regional team. Of the 49 districts, 45 had a Sangini pharmacy with a TSV performed in the last 2 years; thus, the target population was pharmacies in these districts. Within each of the supervision areas, we selected 4 districts using probability proportional to size sampling with replacement, where the population size was the number of eligible Sangini pharmacies in the district. Wherever possible, we selected a buffer of additional pharmacies (up to 20% more than needed in each district) as replacements in case pharmacies declined to participate in the survey or the mystery clients could not locate selected pharmacies. However, this was not possible in all districts due to the low number of Sangini pharmacies. The sample size was calculated based on a detectable difference of 10% with level of confidence (alpha) of 5% and power (beta) of 80%. All pharmacies were contacted at least 2 weeks before the start of data collection to obtain their consent to participate in the mystery client survey.

Mystery clients were young women trained as interviewers to visit selected pharmacies posing as clients who are interested in starting to use a contraceptive method. To examine Sangini provider behavior under different circumstances, the survey included 2 scenarios: (1) the mystery client approached the pharmacy without a specific contraceptive method in mind and asked the provider for a recommendation; and (2) the mystery client told the provider that she would like to use oral contraceptives. Because there was only 1 mystery client visit per facility, the scenario to be used was randomly assigned for each facility.

In both scenarios, the mystery client said that she is married. If asked, she said that she has 1 child who is aged 1 year and that she would like to have more children in the future but wants to wait several years before her next birth as she and her husband are in the process of moving so that he can find work. This client profile was designed to steer the provider toward recommending one of the short-acting contraceptive methods that CRS supplies to Sangini outlets: injectable contraceptives, oral contraceptives, or condoms. During their visits, the mystery clients carefully observed the outlet and the provider to determine whether they followed guidelines for FP counseling and recommended a contraceptive method that was appropriate for the client profile. After the pharmacist recommended a method and offered it to the client, the mystery clients were instructed to say they would think about it and leave the shop.

Each mystery client traveled with an interviewer who helped to locate the pharmacy and administered the post-visit questionnaire in a private place immediately after the mystery client left the pharmacy. The survey used 1 questionnaire for both scenarios. The questionnaire asked about visit length, provider greeting, privacy, presence of CRS information, education, and communication materials, pharmacy cleanliness, FP-related screening questions asked by the provider, method information given by the provider (how to use, effectiveness, side effects, advantages, and disadvantages), and which method(s) the provider recommended.

SHOPS Plus recruited experienced enumerators to act as mystery clients and interviewers by drawing from its contracted local research firm's enumerators pool. The majority of mystery clients and interviewers had completed their Bachelor's degree. Mystery clients had to look between ages 22 and 26 years, but caste and ethnicity were not considered. Mystery clients and interviewers participated in a 5-day training before data collection. For 3 days, they attended classroom trainings, which covered topics including roles and responsibilities, research ethics, study methodology, both of the mystery client scenarios, role play, logistics, and use of the electric platform for data collection. They also participated in 2 days of pilot testing in the field, which provided them with the opportunity to practice the mystery client scenarios and discuss any issues. The contractor trained approximately 30% more mystery clients than needed for final data collection and hired the mystery clients who performed best during training and piloting to conduct final data collection.

### Comparing TSV and Mystery Client Visit Data

TSVs are led by specially trained CRS quality assurance (QA) officers. The purpose of these visits is to monitor and improve the quality of FP services provided by Sangini outlets. During a TSV, the QA officer observes the facility and, ideally, observes interactions between service providers and FP clients. However, because it is often not possible to observe the provider interacting with a client at the time of the TSV, the QA officer frequently uses vignettes to elicit a hypothetical experience to evaluate the provider's clinical FP knowledge and skills. To do this, QA officers read a script that presents a specific client situation and asks the provider to say and do everything they would with a client when providing FP. The script covers multiple scenarios, including a new client asking for a recommendation for a method, a new client who wants to start a specific method (injectables, oral contraceptives, condoms, or emergency contraceptives) and a returning client who wants to use the same method. QA officers use a visit checklist to guide their evaluation of the provider's performance. Notably, the script prompts providers if they miss a step in the counseling process. For instance, if a provider is at the stage of a visit where they should screen for pregnancy, the QA officer will ask the provider to tell and show them what they would do next for the client. If the provider does not say that they would screen for pregnancy, the QA officer will ask them specifically how they would screen for pregnancy. If the provider then correctly describes how to screen for pregnancy, the QA officer records that the provider has successfully completed this task. After going through the checklist, QA officers share feedback with the provider and develop an action plan for identified areas of improvement. Facilities with the lowest TSV scores are prioritized for return TSVs and refresher trainings for service providers.

The mystery client visit data were merged with the routine data collected during TSVs to Sangini outlets, using unique IDs of the same Sangini outlets. Consequently, data were compared for the same 414 facilities but not necessarily the same providers. Although data on the number of providers per facility were not available, most rural outlets only have 1 provider. The analysis used the latest TSV data if there were more than 1 TSV available for an outlet. The analyses only used variables that were similarly measured in both the mystery client visits and TSVs. However, as the TSV checklist was revised in 2018, approximately half of the TSVs we analyzed were conducted using the previous version of the checklist. Consequently, although we compared the same 414 facilities, the TSV denominator for certain questions was less than 414 because the previous version of the TSV checklist did not have a comparable variable to the mystery client survey for those questions. The mystery client denominator for counseling on oral contraceptives and injectables is also slightly less than 414 due to systematic skipping. Lastly, because TSVs are designed as coaching tools and allow for prompting, we anticipated *a priori* that scores would be higher from the hypothetical TSV data than from the real-life mystery client data.

For this analysis, data collected during TSVs were considered “knowledge,” data collected during mystery client visits were considered “practice,” and the difference between the 2 is an estimate of the “know-do gap” among Sangini providers. The significance of this difference was calculated using paired t-tests, with the pairing representing the data from the mystery client visits and the TSVs within the same pharmacy. A threshold of *P*<.05 is used to determine statistical significance. Data were analyzed using Stata 16 and linearized standard errors to account for the survey design.^21^

### Ethics Approval

The Abt Associates Institutional Review Board provided ethical approval for the study on August 7, 2018, and the Nepal Health Research Council Ethical Review Board provided ethical approval on August 15, 2018. Data were transferred and submitted by the research firm through the web-based platform Huddle. All data sets were destroyed by the research firm after SHOPS Plus provided the final approval of the clean dataset.

## RESULTS

Mystery clients visited 422 Sangini outlets and ultimately surveyed 414 outlets that had received at least 1 TSV in the preceding 2 years.

### Privacy

When a pharmacy client asks about FP or an FP method, Sangini providers are trained to offer the client privacy for FP counseling and/or administration and tell the client that her personal information will be kept confidential. During TSVs, more than three-fourths (77.4%) of providers reported they would offer the client privacy for FP counseling, but less than one-fourth (16.2%) actually did so in the mystery client visits (*P*<.001). The majority of providers (89.4%) also said they would assure clients of confidentiality during the TSVs, compared to 3.4% who assured mystery clients (*P*<.001) (Table 1).

**TABLE 1.** Technical Support Visit and Mystery Client Visit Data Comparison: Privacy and Confidentiality

	Mystery Client Visit (N=414)	Technical Support Visit (N=410)	Know-Do Gap
	% (95% Confidence Interval)	% (95% Confidence Interval)	% (95% Confidence Interval)
Provider offered the client privacy for family planning counseling and administration	16.2 (13.7, 19)	77.4 (74.3, 80.2)	61.0^a^ (57.1, 65.0)
Assured confidentiality	3.4 (2.6, 4.4)	89.4 (87.7, 90.8)	86.0^a^ (84.2, 87.7)

a *P*<.001; *P*-values calculated using t-test on paired values; N is the number of observations.

### Client Needs Assessment and Eligibility

Sangini providers are trained to ask all potential FP clients the list of questions in Table 2 and take their blood pressure to assess their FP needs and determine medical eligibility for hormonal methods. There is a highly statistically significant difference (*P*<.001) between mystery client visits and TSVs for all 8 indicators required to assess client needs and determine their eligibility for hormonal FP methods (Table 2). Starting with the smallest gap, most providers both in TSVs (78.3%) and mystery client visits (67.7%) asked/said they would ask clients if they were currently using any FP methods, with a 12.2 percentage point gap between these results. Very few providers, both in TSVs (34.6%) and mystery client visits (6.8%) asked or said they would ask in vignettes if the client was postpartum. More than half of providers asked clients when they had their last period in mystery client visits (56.5%), compared to almost 100% in TSVs (95.1%). During TSVs, 84.6% of providers—double the percentage of providers in mystery client visits (41.7%)—said they would ask clients about their plans for having additional children. There was a gap of between 60 and 80 percentage points for TSV and mystery client visit results for 3 indicators: saying they would ask versus asking if the client has abnormal breast lumps or breast cancer (TSV: 76.5%, mystery client: 12.7%); if the client has unexplained vaginal bleeding (TSV: 82.0%, mystery client: 8.2%); and if the client is currently taking any medications (TSV: 92.7%, mystery client: 24.3%). Finally, there was a gap of more than 90 percentage points between TSV and mystery client visit results for whether the provider said they would take a hypothetical client's blood pressure, with under 4% of providers completing this step in the mystery client visits (TSV: 94.1%, mystery client: 3.8%). Results varied minimally when broken out by mystery client scenario type; see Supplement for this breakdown.

**TABLE 2.** Technical Support Visit and Mystery Client Visit Data Comparison: Assessing Client Needs and Eligibility

	Mystery Client Visit		Technical Support Visit		Know-Do Gap
	% (95% Confidence Interval)	N	% (95% Confidence Interval)	N^a^	% (95% Confidence Interval)
Client needs					
Currently using any FP methods	67.7 (64.6, 70.7)	414	78.3 (74.8, 81.5)	187	12.2^b^ (6.6, 17.8)
Postpartum	6.8 (5.8, 8.0)	414	34.6 (30.4, 39.1)	187	23.9^b^ (19.0, 28.8)
Plans for additional children	41.7 (38.6, 44.9)	414	84.6 (81.9, 86.9)	409	43.2^b^ (38.9, 47.4)
Medical eligibility					
Time of last menstrual period	56.5 (53.1, 59.8)	414	95.1 (92.5, 96.9)	187	36.8^b^ (31.6, 42.1)
Abnormal breast lumps or breast cancer	12.7 (11.0, 14.7)	414	76.5 (73.7, 79.1)	408	63.7^b^ (60.5, 67.0)
Unexplained vaginal bleeding	8.2 (6.9, 9.7)	414	82.0 (79.2, 84.5)	409	73.9^b^ (70.8, 76.9)
Currently taking any medication	24.3 (22.0, 26.7)	414	92.7 (89.9, 94.8)	223	77.9^b^ (74.3, 81.5)
Took blood pressure	3.8 (2.7, 5.2)	414	94.1 (92.7, 95.3)	408	90.3^b^ (88.5, 92.0)

a The number of observations varies for the TSV data because the TSV checklist was revised during the period in which TSVs analyzed for this study were collected. Consequently, although we compared the same 414 facilities, the TSV denominator for certain questions was less than 414 because the previous version of the TSV checklist did not have a comparable variable to the mystery client survey for those questions.

b *P*<.001; *P*-values calculated using t-test on paired values; N is the number of observations.

There is a highly statistically significant difference between mystery client visits and TSVs for all 8 indicators required to assess client needs and determine their eligibility for hormonal FP methods.

### Counseling on Injectable Contraceptives

There was not a statistically significant difference in the providers' counseling on injectables during mystery client visits and TSVs for mentioning side effects or for what to do if a client has side effects (*P*=.52 and *P*=.41, respectively), with between 93% and 94% of providers having counseled on these topics in mystery client visits and TSVs (Table 3). In TSVs, nearly all (98.9%) providers explained how the method is used, compared to 83.8% of providers during mystery client visits (*P*<.001). Providers were more likely to discuss the advantages (*P*<.001) and slightly more likely to discuss disadvantages (*P*=0.05) of this method in TSVs (96.4% and 97.5%, respectively) than in mystery client visits (63.4% and 95.2%, respectively). Overall levels of counseling are high—more than 80%—in both TSV and mystery client visits.

**TABLE 3.** Technical Support Visit and Mystery Client Visit Data Comparison: Counseling on Injectable Contraceptives

	Mystery Client Visit		Technical Support Visit		Know-Do Gap
	% (95% Confidence Interval)	N	% (95% Confidence Interval)	N^a^	% (95% Confidence Interval)
Provider explained how to use this method	83.8 (81.9, 85.5)	401	98.9 (98.0, 99.5)	186	16.4^b^ (10.3, 22.5)
Provider mentioned the side effects of this method	93.8 (92.1, 95.1)	401	93.3 (91.8, 94.5)	409	−0.6 (−2.6, 1.3)
Provider mentioned what to do if you experience side effects while using this method	94.0 (92.4, 95.3)	401	93.3 (91.7, 94.7)	409	−0.8 (−2.8, 1.2)
Provider mentioned advantages of method	63.4 (60.6, 66.1)	401	96.4 (94.6, 97.6)	223	37.8^b^ (33.4, 42.2)
Provider mentioned disadvantages of this method	95.2 (93.7, 96.3)	401	97.5 (95.7, 98.6)	223	2.4^c^ (0.1, 4.8)

a The number of observations varies for the TSV data because the TSV checklist was revised during the period in which TSVs analyzed for this study were collected. Consequently, although we compared the same 414 facilities, the TSV denominator for certain questions was less than 414 because the previous version of the TSV checklist did not have a comparable variable to the mystery client survey for those questions.

b *P*<.001; *P*-values calculated using t-test on paired values; N is the number of observations.

c *P*<.05.

### Counseling on Oral Contraceptives

Similar to results for injectables, levels of counseling were generally high for all indicators except explaining the advantages of the method, in both TSVs and mystery client data (Table 4). There is a highly statistically significant difference (*P*<.001) for all 5 oral contraceptive counseling indicators between mystery client visits and TSVs. Nearly all providers explained how to use oral contraceptives in TSVs (98.3%) whereas 82.7% of providers in mystery client visits explained their use. Similarly, nearly all providers explained what to do if there are side effects in TSVs (98.9%) whereas 86.8% of providers in mystery client visits explained what to do. However, providers were more likely to mention the side effects of oral contraceptives and the disadvantages of oral contraceptives during mystery client visits (96.0% and 94.2%, respectively) than during TSVs (82.9% and 77.7%, respectively) but were more likely to mention the advantages of oral contraceptives during TSVs (67.4%) than in MC visits (50.4%).

**TABLE 4.** Technical Support Visit and Mystery Client Data Comparison: Counseling on Oral Contraceptives

	Mystery Client		Technical Support Visit		Know-Do Gap
	% (95% Confidence Interval)	N	% (95% Confidence Interval)	N^a^	% (95% Confidence Interval)
Provider explained how to use this method	82.7 (80.6, 84.5)	406	98.3 (97.1, 99.0)	373	15.7^b^ (13.4, 18.0)
Provider mentioned the side effects of this method	96.0 (94.5, 97.2)	406	82.9 (80.6, 85.0)	411	−13.2^b^ (−15.9, −10.6)
Provider mentioned what to do if you experience side effects while using this method	86.8 (84.6, 88.8)	406	98.9 (98.3, 99.3)	398	12.3^b^ (10.1, 14.5)
Provider mentioned advantages of this method	50.4 (47.3, 53.4)	406	67.4 (62.9, 71.5)	223	27.7^b^ (20.9, 34.5)
Provider mentioned disadvantages of this method	94.2 (92.5, 95.5)	406	77.7 (73.6, 81.3)	223	−16.6^b^ (−21.0, −12.1)

a The number of observations varies for the TSV data because the TSV checklist was revised during the period in which TSVs analyzed for this study were collected. Consequently, although we compared the same 414 facilities, the TSV denominator for certain questions was less than 414 because the previous version of the TSV checklist did not have a comparable variable to the mystery client survey for those questions.

b *P*<.001; *P*-values calculated using t-test on paired values.

## DISCUSSION

To our knowledge, this is the first study that examines the quality of counseling provided by pharmacists licensed to provide injectable contraception that compares their knowledge (i.e., how they perform when they know they are being observed) to how they perform in practice. Findings from this study highlight know-do gaps in quality FP service provision, especially related to the provision of privacy and determining client needs and medical eligibility. Encouragingly, this study provides evidence that a large majority of pharmacists can and do knowledgeably counsel women about pharmacist-administered injectables and oral contraceptives.

To our knowledge, this is the first study that examines the quality of counseling provided by pharmacists licensed to provide injectable contraception that compares their knowledge to how they perform in practice.

The know-do gap in provision of privacy for FP counseling and assured confidentiality may impact the large know-do gap identified among several client needs and eligibility assessment indicators, especially if the provider is male. Indeed, 75% of Sangini providers are men. As providers are, in practice, not providing privacy for FP counseling approximately 84% of the time (as seen by the mystery client visit result of 16.2% providing privacy), they may not feel comfortable asking female clients more intimate questions in a public space or may anticipate that female clients would not feel comfortable answering due to embarrassment among others in the pharmacy. These questions may include if the client has had unexplained vaginal bleeding, a question that was asked in only 8.2% of mystery client visits (representing a 73.9 percentage point gap with TSV results). Of the mystery clients that were offered privacy, nearly double the percentage (15%) were asked this question. While this percentage is still low, this does suggest that addressing the know-do gap for ensuring privacy may, in turn, reduce the know-do gap for these indicators.

Moreover, the Sangini training did not adequately emphasize the importance of assessing FP clients' medical eligibility, other than taking blood pressure. In addition, many providers reported that they did not have the time to complete all these steps while running their pharmacies. Looking at the gap in taking blood pressure specifically (90.3%), there may have been a perception among the providers that for young clients, blood pressure was not typically an issue and thus taking blood pressure was not a priority. Although the mystery client survey suggests that this is a critical gap to address, it is important to note that the WHO emphasizes that providers not withhold a method if unable to take blood pressure, as the methods that Sangini outlets provide are very low risk.^19^

Similarly, considering the major gap in assessing medical eligibility for mystery clients who would like to start oral contraceptives (Supplement), it is important to note that although the Nepalese Ministry of Health and Population encourages medical screening for oral contraceptives, this method is provided over the counter in Nepal. Therefore, it is not required for providers to ask any medical eligibility or needs assessment questions before selling the product. Indeed, the Ministry notes that the health benefits of oral contraceptives “far exceed their health risk.”^22^ Consequently, closing the gap in assessing medical eligibility for clients seeking oral contraceptives may be less of a priority as compared to addressing other findings identified through this study. In addition, some of the medical eligibility criteria analyzed are only relevant for certain methods.^19^ If a client comes in with a method selected, it may be appropriate for the Sangini providers to only assess medical eligibility criteria relevant to that method. However, if a client has not selected a method before her visit, it may be preferred to ask all medical eligibility criteria questions before counseling.

Overall, the findings suggest that providers are doing very well adhering to quality standards of counseling on oral contraceptives and injectables, with a high percentage of providers completing the required steps in both TSVs and mystery client visits. Moreover, the percentage of providers in the mystery client visits that completed each counseling step was much higher overall as compared to those completing steps for the provision of privacy and assessing client needs and eligibility. Consequently, although providers have room to improve on the provision of privacy and assessing client needs and eligibility, they are providing their clients with the information needed to select a method most appropriate for them.

Providers have room to improve on providing clients with privacy and assessing client needs and eligibility but are providing clients with the information they need to choose an appropriate method.

As Sangini is the first and only private pharmacy network in Nepal approved to offer injectables, it is promising to see that counseling for this method is similar to that of oral contraceptives. In fact, for 4 of the 5 counseling indicators, providers performed better on counseling for injectables as compared to oral contraceptives for both mystery client visits and TSVs. These data suggest that providers are doing just as well and potentially better counseling on injectables than oral contraceptives.

Some of the gaps highlighted in this study are similar to those found in other mystery client surveys. A mystery client survey of private and public FP providers in India found that providers rarely asked about clients' medical history. However, privacy was maintained (no other clients or providers in the room) in 25 of the 40 (62.5%) interactions documented at private facilities, which is a higher outcome than the 3.4% seen at the Sangini clinics in this study.^23^ Still, Sangini providers were very strong in counseling on both oral contraceptives and injectables as compared to the study in India. Only in 13% of the mystery client visits in the India study did the provider describe the potential side effects of the prescribed method without being asked by the mystery client. Moreover, providers rarely discussed management of side effects if encountered,^23^ while more than 80% of providers in this study did both. Similarly, in a mystery client study that was conducted before and after training and supportive supervision of pharmacists in Vietnam, only 54 of 216 pharmacists provided information on side effects of emergency contraceptive pills post-intervention.^24^ These data may suggest that Sangini providers counseled better on side effects than is typically seen in other mystery client surveys.

With many countries considering expanding the role of pharmacies in FP service provision, especially to provide injectables, there is a growing need to explore models like the Sangini network and ways to use supportive supervision and training to improve service quality. Using these research findings, CRS redesigned its Sangini training curriculum to streamline lectures and increase focus on competency-based learning. CRS also retrained all of its QA officers on how to properly conduct a TSV in the field. Other programs should consider a strong emphasis on hands-on learning to support providers in establishing the practical skills needed to properly conduct a client visit. In addition, CRS altered the way that TSVs are scored to ensure the comprehensive and accurate evaluation of provider skills. It is important to review scoring practices to minimize unintentional bias that overstates provider capabilities to best determine and address weaknesses in quality service delivery.

With many countries considering expanding the role of pharmacies in FP service provision, especially to provide injectables, there is a growing need to explore models like the Sangini network and ways to use supportive supervision and training to improve service quality.

The study findings also suggest that supervision alone does not necessarily ensure full adherence to protocols; consequently, it is necessary to conduct monitoring of supervision to better understand potential quality issues. Mystery client surveys are one way to do this, but there are several additional options. For instance, in sub-Saharan Africa, Marie Stopes International supplements supportive supervision visits with random spot checks, an anonymous complaint telephone line for clients, and audits.^25^ In addition, a study measuring the efficacy of supervision on community health workers in Mozambique completed a series of in-depth interviews with health workers, health facility supervisors, district health worker supervisors, and community leaders. These interviews focused on understanding the impact of supervision on health worker motivation and program implementation. In this way, the study identified barriers to supervision from multiple perspectives, thereby providing a more comprehensive view of the situation.^26^ Therefore, there are several options for monitoring supportive supervision visits, including mystery client surveys, that may be useful for CRS and other organizations implementing pharmacy networks to use to measure the utility of these visits. Further research into cost-effective and routine means of ensuring quality and quality supervision is needed.

### Methodological Considerations

Mystery client surveys are becoming an increasingly popular way to evaluate FP service quality and the impact of supportive supervision.^23^^,^^24^ Still, there are concerns around data quality and ethics. Earlier mystery client studies, including a 1985 study in Nepal, encountered challenges in collecting adequate data for multiple reasons. First, mystery clients were recruited informally, meaning that these individuals were not professional enumerators. The Nepal study focused on the quality of services at FP clinics for lower-caste and low-caste women; however, several of these women were so afraid of the clinics that they dropped out of the study, which raised ethical concerns and limited data collection. Mystery clients should know what to expect when they go into clinics to reduce fear, and qualitative research experts should be available to provide guidance and support to mystery clients in the field. In addition, clients in the Nepal study were asked to recall their experience at the clinic in detail, rather than respond to a list of predetermined questions to measure service quality. Lastly, top managers of facility systems were informed about the study and agreed to participate, but staff at clinics who interacted with clients were not aware that the study was taking place.^27^ More recent mystery client studies have taken steps to increase data reliability. A 2014 mystery client study in Kenya used an objective checklist to discuss provider quality with clients and held a weeklong training session with role play and pilot testing. Still, mystery clients may not be able to fully replicate the actions of actual clients, which may impact provider behavior.^28^ In 2017, Fitzpatrick and Tumlinson detailed best practices for mystery client studies, which include the steps noted above as well as additional guidance, such as recruiting mystery clients with strong recall ability and standardizing mystery client behavior to allow for comparison across visits. Despite the challenges that come with implementing mystery client studies, they are still seen as a valuable tool to collect data about service quality that is unavailable through other research methods.^29^ The methodology used by SHOPS Plus reflects these learnings to mitigate problems encountered in past mystery client studies.

### Limitations

We note that because pharmacies were contacted by phone 2 weeks before the mystery client visits for informed consent, as required by the local Ethics Review Board, it is possible that the providers recognized the mystery clients as such. If this occurred, providers may have been on their “best behavior” to counsel clients in accordance with their training. This could have possibly biased our mystery client results to be overly positive.

## CONCLUSION

Private pharmacies such as those in the Sangini network provide a promising opportunity to increase access to injectables. Indeed, this study provides evidence that pharmacists in Nepal are able to adhere very well to quality standards on counseling for injectables, as well as oral contraceptives. However, some shortcomings remain, with findings demonstrating a highly significant know-do gap among Sangini providers in the provision of privacy and determining clients' needs and medical eligibility. Providers know what is required to ensure a high-quality client experience, but they may not understand the value of or have the time to conduct all the necessary steps. The concrete measures CRS took to narrow this gap may provide useful guidance for other countries expanding the role of pharmacies in FP service provision. Still, findings suggest that supervision alone does not necessarily ensure full adherence to protocols; consequently, further research into cost-effective and routine means of ensuring quality and quality supervision is needed. Although findings highlighted several areas for improvement, the fact that Sangini providers counseled similarly for the provision of oral contraceptives and injectables is a positive indicator that pharmacists can successfully expand their FP offerings and equip clients with the information needed to select a method of their choice.

## Supplementary Material

GHSP-D-21-00657-supplement.pdf

## Footnotes

* DHS collects data for socially marketed pill and condom brands only, not injectables. It does collect data on the percentage of injectable users that obtain their method from private pharmacies. We have included this statistic with the assumption that only private pharmacies that are licensed to sell injectables (thereby Sangini pharmacies only) are selling injectables, and that there is no illegal selling of injectables by nonlicensed private pharmacies.

## Notes

**Peer Reviewed**

**First published online:** May 20, 2022.
