# Supplementary material for: The Know-Do Gap: Understanding and Improving Service Quality Among Pharmacies Providing Injectable Contraceptives Through a Mystery Client Study in Nepal
Source: Glob Health Sci Pract. 2022 Jun 29;10(3):e2100657. doi: 10.9745/GHSP-D-21-00657 (PMC9242610; doi:10.9745/GHSP-D-21-00657)
Supplement: GHSP-D-21-00657-supplement.pdf [file GHSP-D-21-00657-supplement.pdf]

Supplement to: Karki S, Chappell M, Johns B, Bradley SEK. The know-do gap: Understanding and improving service quality among pharmacies providing injectable contraceptives through a mystery client study in Nepal. *Glob Health Sci Pract*. 2022;10(3):e2100657. https://doi.org/10.9745/GHSP-D-21-00657

Supplement Table 1: TSV and Mystery client scenario 1 (ask provider for recommendation on contraceptive method) comparison: Assessing client needs and eligibility

Variables	Mystery client			Technical Support visit			Know-do gap	
	%	Confidence interval	N	%	Confidence interval	N[1]		Confidence interval
Client Needs								
Currently using any FP methods	69.2	63.6-74.4	206	79	71.4-85	92	13.6*	2.2-25
Postpartum	7.3	5.2-10.1	206	36.6	28.4-45.6	92	26.6***	16.6-36.7
Plans for additional children	43	37.4-48.9	206	85.5	80.6-89.3	205	42.3***	34.6-50.1
Medical Eligibility								
Time of last menstrual period	55.1	49-61.1	206	96.4	92-98.4	92	37.3***	28.3-46.3
Abnormal breast lumps or breast cancer	10.5	7.7-14.3	206	76.9	71.8-81.3	204	66.3***	60.4-72.1
Unexplained vaginal bleeding	8.1	5.6-11.5	206	82.2	76.9-86.5	204	74***	68.3-79.7
Currently taking any medication	19.6	15.7-24.2	206	94.2	89.4-96.9	113	84.3***	79-89.7
Took blood pressure	3.7	2.2-6.3	206	95.4	92.7-97.1	204	91.7***	88.7-94.6

** = p<0.05, ** = p<0.01, ***p<0.001; p-values calculated using t-test on paired values; N is the number of observations.*

[1] The number of observations varies for the TSV data because the TSV checklist was revised during the period in which TSVs analyzed for this study were collected. Consequently, although we compared the same 414 facilities, the TSV denominator for certain questions was less than 414 because the previous version of the TSV checklist did not have a comparable variable to the mystery client survey for those questions. N of TSVs is split between Tables 1 and 2 to compare more accurately against the N of the two scenarios.

Supplement to: Karki S, Chappell M, Johns B, Bradley SEK. The know-do gap: Understanding and improving service quality among pharmacies providing injectable contraceptives through a mystery client study in Nepal. *Glob Health Sci Pract*. 2022;10(3):e2100657. https://doi.org/10.9745/GHSP-D-21-00657

Supplement Table 2: TSV and Mystery client scenario 2 (ask provider for oral contraceptives) comparison: Assessing client needs and eligibility

Variables	Mystery client			Technical Support visit			Know-do gap	
	%	Confidence interval	N	%	Confidence interval	N[2]		Confidence interval
Client Needs								
Currently using any FP methods	66.3	60.4-71.7	208	77.7	70.4-83.7	95	10.9	-0.1-21.8
Postpartum	6.4	4.4-9.1	208	32.8	25.2-41.6	95	21.3***	11.5-31.2
Plans for additional children	40.5	34.8-46.4	208	83.7	78.5-87.8	204	44***	36.3-51.6
Eligibility								
Time of last menstrual period	57.9	51.7-63.8	208	93.9	87.3-97.2	95	36.4***	25.7-47.1
Abnormal breast lumps or breast cancer	14.8	11.3-19.2	208	76.1	70.6-80.8	204	61.3***	55-67.6
Unexplained vaginal bleeding	8.3	5.8-11.7	208	81.8	76.5-86.2	205	73.7***	68.2-79.3
Currently taking any medication	28.8	24.2-33.8	208	91.2	85-95	110	71.5***	64.1-78.9
Took blood pressure	3.9	2-7.3	208	92.9	89.9-95	204	89***	85.5-92.4

** = p<0.05, ** = p<0.01, ***p<0.001; p-values calculated using t-test on paired values; N is the number of observations.*

[2] The number of observations varies for the TSV data because the TSV checklist was revised during the period in which TSVs analyzed for this study were collected. Consequently, although we compared the same 414 facilities, the TSV denominator for certain questions was less than 414 because the previous version of the TSV checklist did not have a comparable variable to the mystery client survey for those questions. N of TSVs is split between Tables 1 and 2 to compare more accurately against the N of the two scenarios.
